# Sirenomelia Sympus Dipus: Autopsy Case Report of a Second-Trimester Fetus With Multiple Congenital Anomalies

**DOI:** 10.7759/cureus.110114

**Published:** 2026-06-02

**Authors:** Gustavo A Cantú Perches, Paola P Pulido Puente, Luis A Lugo Morán

**Affiliations:** 1 Legal and Forensic Medicine, University Hospital "Dr. José Eleuterio González", Monterrey, MEX; 2 Pediatrics, University Hospital "Dr. José Eleuterio González", Monterrey, MEX; 3 Forensic Medicine, Autonomous University of Nuevo León, Monterrey, MEX

**Keywords:** congenital anomalies, fetal autopsy, lower limb fusion, mermaid syndrome, sirenomelia, sympus dipus

## Abstract

Sirenomelia (mermaid syndrome) is an extremely rare and lethal congenital malformation characterized by fusion of the lower limbs, with a global incidence of approximately one in 60,000-100,000 live births. No proven hereditary pattern or genetic basis has been established. Sympus dipus (Type I, Stocker-Heifetz classification) is the variant in which both feet are preserved despite lower-limb fusion. We report the autopsy findings of a 19-week male fetus received at the Department of Anatomical Pathology of Hospital Metropolitano "Dr. Bernardo Sepúlveda" in Monterrey, México. External examination confirmed complete lower-limb fusion with two preserved feet and a lumbosacral myelomeningocele. Internal examination revealed transposition of the great vessels, absent renal arteries from the abdominal aorta, bilateral renal hypoplasia, agenesis of the bladder, ureters, and urethra, Bochdalek diaphragmatic hernia with bilateral pulmonary hypoplasia, bilobed right lung, pyloric and duodenal atresia, rectal atresia, imperforate anus, multiple subcapsular hepatic hematomas, and bilateral cryptorchidism. Radiography confirmed sacral and pelvic agenesis with two femora, tibiae, and feet. The placenta showed a two-vessel umbilical cord. Findings broadly correlate with the existing literature on sirenomelia; however, Bochdalek diaphragmatic hernia and bilobed right lung appear to represent novel associated anomalies not previously reported for sympus dipus.

## Introduction

Sirenomelia, commonly known as mermaid syndrome, is an extremely rare and lethal congenital malformation characterized by the fusion of the lower limbs [1]. Its reported incidence ranges from one in 60,000 to one in 100,000 live births globally, with no proven hereditary pattern or established genetic basis [1,2]. Approximately 300 cases have been reported in the world literature [1,2].

The Stocker-Heifetz classification divides sirenomelia into three types based on the degree of lower limb fusion and the presence of feet: sympus dipus (Type I, both feet present), sympus monopus (Type II, one foot present), and sympus apus (Type III, no feet present) [2]. Associated anomalies commonly include genitourinary agenesis, sacral and pelvic bone agenesis, gastrointestinal malformations, and cardiovascular defects [3,4].

The pathogenesis remains controversial, with two main theories proposed: the vascular steal theory, which attributes the malformation to an aberrant vitelline artery diverting blood flow away from the caudal structures [5,6], and the blastogenesis defect theory, which proposes a primary defect in the caudal mesoderm during early embryonic development [7]. Maternal risk factors include diabetes mellitus [8,9].

A narrative literature review was conducted using PubMed, Google Scholar, and Scopus databases. Search terms included "sirenomelia," "sympus dipus," "mermaid syndrome," "fetal autopsy," and "congenital anomalies." All available case reports, case series, and review articles in English were considered. The review focused on identifying previously reported anatomical findings associated with the sympus dipus variant to contextualize the anomalies documented in the present case.

We present the autopsy findings of a 19-week male fetus with sympus dipus and multiple associated congenital anomalies, including findings not previously identified in the reviewed literature in association with this subtype [10,11].

## Case presentation

A second-trimester male fetal product of approximately 19 weeks of gestational age was received at the Department of Anatomical Pathology of Hospital Metropolitano "Dr. Bernardo Sepúlveda" in Monterrey, Nuevo León, México, in 2017. The product was referred for academic autopsy with radiographic evaluation, with the objective of documenting internal malformations and comparing findings with the national and international literature. Maternal clinical records were not available for review.

**Figure 1:**
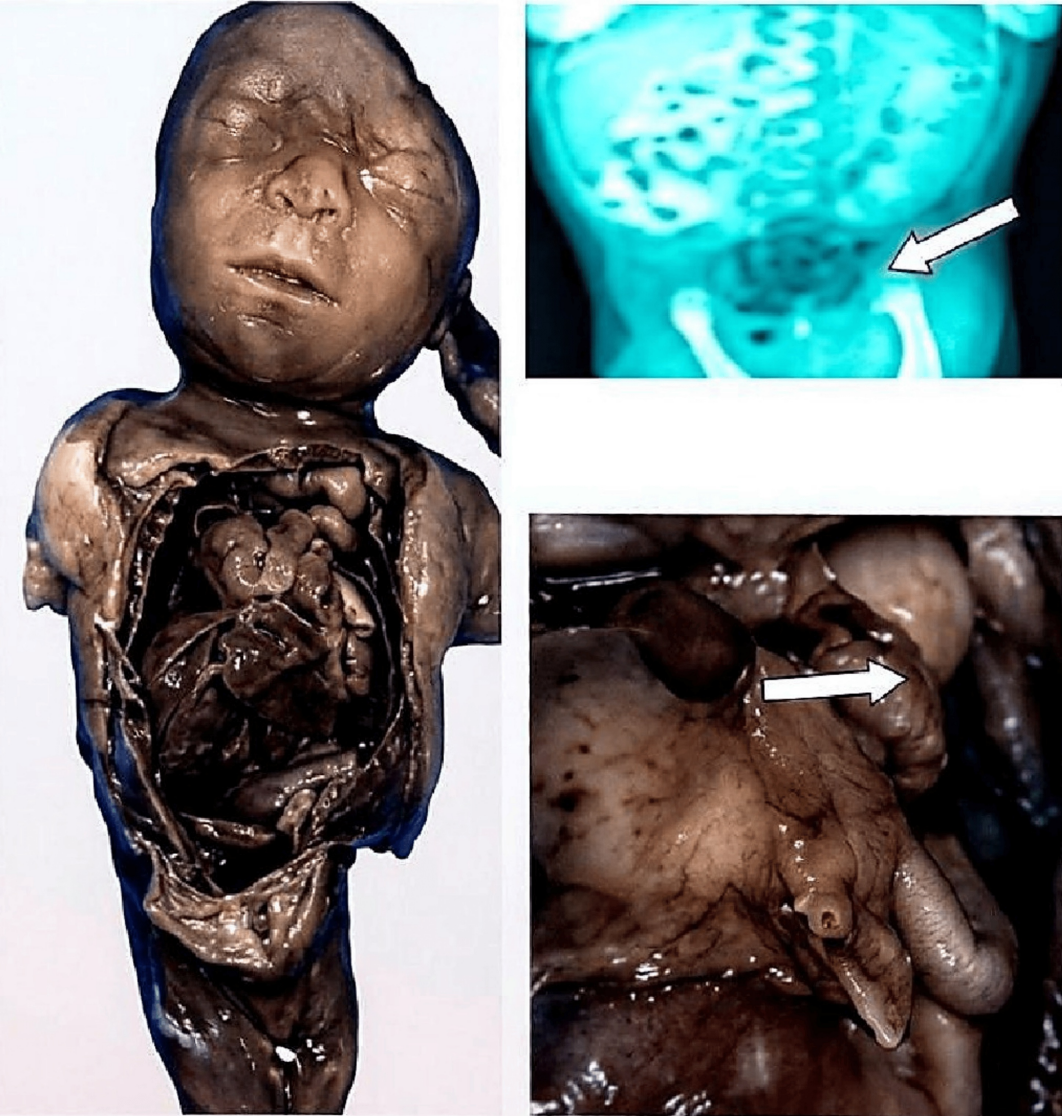
Autopsy composite. Left: anterior view of the fetus following thoraco-abdominal opening, showing the eviscerated cavity with multiple visceral anomalies including intestinal loops and cardiovascular structures. Upper right: radiographic close-up of the pelvic region demonstrating sacral and pelvic bone agenesis (arrow). Lower right: internal detail showing the Bochdalek diaphragmatic hernia defect (arrow) with herniation of abdominal contents into the thoracic cavity.

External examination revealed a crown-to-coccyx length of 17 cm, consistent with approximately 19 weeks of gestational age. Complete fusion of both lower extremities was the most conspicuous external finding, with both feet preserved, morphologically consistent with Type I sirenomelia (sympus dipus) under the Stocker-Heifetz classification (Figure 1). A lumbosacral myelomeningocele was identified. A diminutive phallus was noted externally. No anomalies of the upper extremities, face, or cranial vault were identified on gross inspection.

Systematic internal examination was performed following standard fetal autopsy protocols. Cardiovascular evaluation revealed transposition of the great vessels, with the aorta arising anteriorly from the right ventricle and the pulmonary artery from the left ventricle. The abdominal aorta lacked discernible renal arterial branches bilaterally. Respiratory examination demonstrated bilateral pulmonary hypoplasia secondary to a left-sided Bochdalek diaphragmatic hernia with herniation of abdominal visceral contents into the thoracic cavity. The right lung demonstrated bilobulation with incomplete interlobar separation. Gastrointestinal findings included pyloric and duodenal atresia, marked meconium distension of the transverse colon, rectal atresia, and imperforate anus. Hepatobiliary examination revealed multiple subcapsular hematomas on the hepatic surface. Urogenital evaluation demonstrated bilateral renal hypoplasia with flattened retroperitoneal kidneys and absent renal arteries from the abdominal aorta, along with complete agenesis of the urinary bladder, bilateral ureters, and urethra. Bilateral cryptorchidism was also identified.

**Figure 2:**
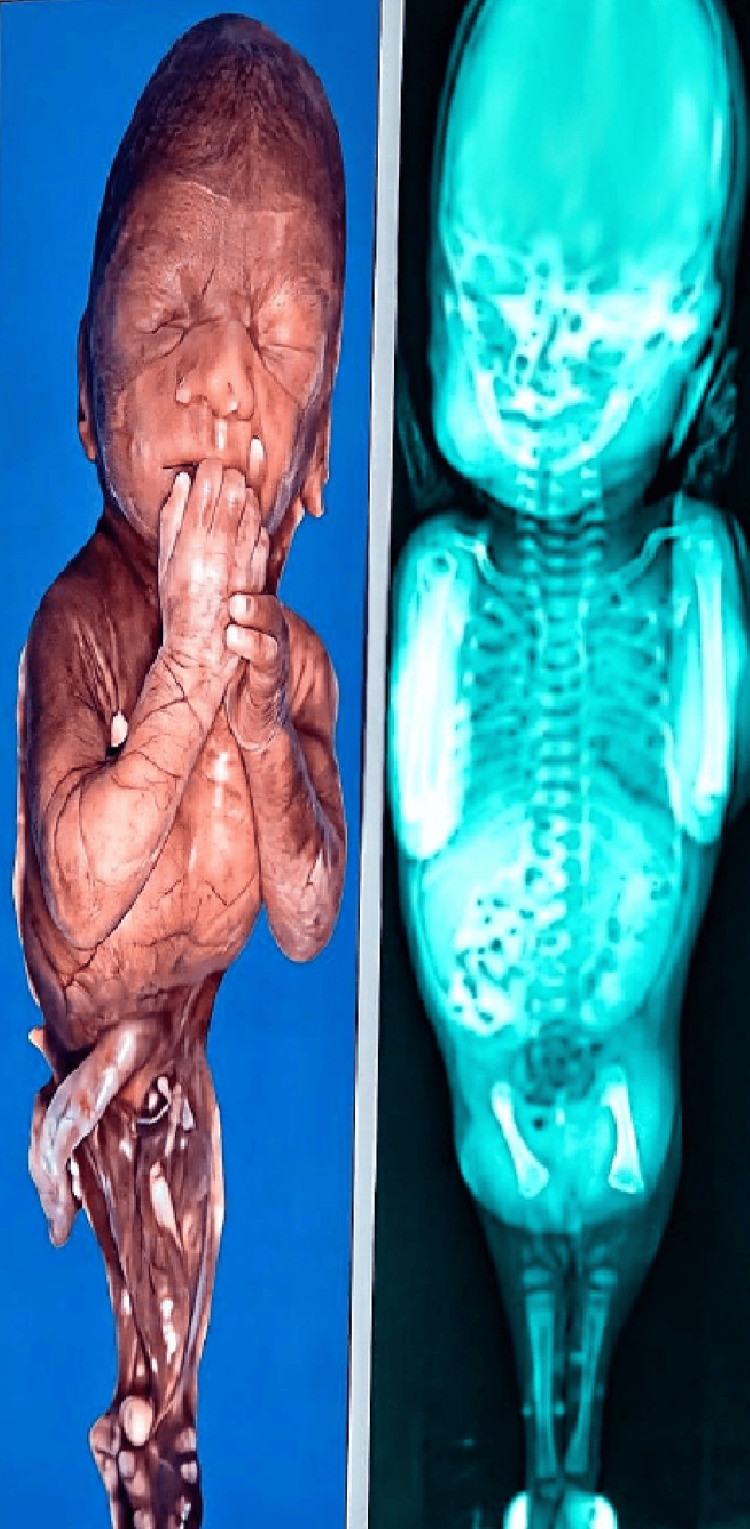
Gross and radiographic findings of sirenomelia sympus dipus in a 19-week male fetus. Left: lateral external gross photograph of the 19-week male fetus demonstrating complete lower-limb fusion (sirenomelia), with both hands positioned at the face, preserved upper extremities, and the characteristic single fused lower extremity with visible feet at the distal end. Right: full anteroposterior fetal radiograph showing an intact cranium, vertebral column, and rib cage; absent sacrum and pelvic bones; and two femora, two tibiae, and two feet within the fused lower extremity — confirming Type I sirenomelia (sympus dipus).

Plain radiographs confirmed agenesis of the sacrum and pelvic bones. Two femora, two tibiae, and two feet were identified within the fused lower extremity, corroborating the sympus dipus classification (Figure 2). Placental examination revealed a two-vessel umbilical cord consisting of a single artery and a single vein, with a second-trimester placenta otherwise within normal limits on macroscopic examination.

​​​​​​The final anatomical diagnoses were: sirenomelia Type I (sympus dipus) with complete lower-limb fusion and two preserved feet; lumbosacral myelomeningocele; transposition of the great vessels; agenesis of sacral and pelvic bones; bilateral renal hypoplasia with absent renal arteries from the abdominal aorta; agenesis of the urinary bladder, ureters, and urethra; left-sided Bochdalek diaphragmatic hernia; bilobed right lung with incomplete interlobar separation; pyloric and duodenal atresia; meconium distension of the transverse colon; rectal atresia; imperforate anus; multiple subcapsular hepatic hematomas; bilateral cryptorchidism; diminutive phallus; and two-vessel umbilical cord with second-trimester placenta. Several of these findings have not been previously identified in association with sympus dipus in the reviewed literature, to our knowledge.

## Discussion

The present case demonstrates a constellation of congenital anomalies consistent with sirenomelia sympus dipus. External lower-limb fusion with preservation of two feet confirmed the Type I classification under the Stocker-Heifetz system, the most favorable anatomical subtype, though the extensive internal involvement rendered this case uniformly lethal [2].

Transposition of the great vessels, as documented here, has been previously described in association with sirenomelia, reinforcing the concept that sirenomelia represents a global caudal developmental field defect rather than an isolated limb anomaly [3]. The absence of renal arteries from the abdominal aorta supports the vascular steal hypothesis: an anomalous umbilical artery arising from the suprarenal aorta is postulated to divert blood flow away from the caudal pole, causing ischemia-mediated dysgenesis of the lower urinary tract, hindgut, and lower extremities [3,5,6].

Renal agenesis or severe hypoplasia constitutes one of the most constant features of sirenomelia and is the primary determinant of its uniform lethality, through the cascade of oligohydramnios and secondary pulmonary hypoplasia [1,2]. The concurrent agenesis of the bladder, ureters, and urethra further illustrates the severity of urinary tract involvement [4]. A two-vessel umbilical cord is also consistently reported and may reflect anomalous persistence of a single vitelline artery [5].

Gastrointestinal anomalies, including anorectal malformations and intestinal atresia, have been previously documented in sirenomelia as part of the caudal mesoderm disruption sequence [4,7]. The pyloric and duodenal atresia in the present case are less commonly reported than anorectal malformations and deserve documentation in future series [8,9].

Of particular interest are findings not previously identified in the reviewed literature in association with the sympus dipus variant, to our knowledge: (1) a Bochdalek diaphragmatic hernia with resultant bilateral pulmonary hypoplasia, and (2) incomplete interlobar separation of the right lung (bilobed right lung). While pulmonary hypoplasia secondary to oligohydramnios is expected in sirenomelia, a concurrent structural diaphragmatic defect provides an additional compounding mechanism [10,11]. The bilobed right lung represents an independent bronchopulmonary anomaly whose co-occurrence with sirenomelia has not been specifically identified in the reviewed literature, to our knowledge [12]. Both findings warrant reporting in future case series.

Placental examination revealed a second-trimester placenta with a two-vessel umbilical cord consisting of a single artery and a single vein, consistent with the vascular steal hypothesis. Macroscopic placental examination did not reveal additional significant abnormalities. Microscopic histopathological examination of the placenta was performed and showed findings consistent with a second-trimester gestation; no significant inflammatory, vascular, or trophoblastic pathology was identified. Formal histopathological examination of fetal organ parenchyma was not performed, which represents a limitation of the present case. Future autopsy protocols for sirenomelia cases should include systematic microscopic examination of all major organ systems to better characterize associated tissue-level anomalies.

Regarding maternal history, no clinical information was available at the time of autopsy referral, including gestational age confirmation, prenatal imaging findings, maternal comorbidities such as diabetes mellitus, or genetic evaluation results. The absence of maternal clinical data represents an additional limitation of this report. Established risk factors for sirenomelia include maternal diabetes mellitus and monozygotic twinning, among others [1,8]; however, these could not be assessed in the present case. Future case reports should systematically document maternal history and prenatal findings to facilitate a better understanding of etiopathogenic factors.

This case contributes to the limited number of systematically documented sirenomelia autopsy cases from México and Latin America. Systematic autopsy reporting from this region is essential for improving regional epidemiological estimates [1,6].

## Conclusions

This case report documents the autopsy findings of a 19-week male fetus with sirenomelia sympus dipus and an extensive constellation of associated congenital anomalies. The core findings - complete lower-limb fusion with two preserved feet, sacropelvic agenesis, bilateral renal hypoplasia with absent renal arteries, agenesis of the bladder, ureters, and urethra, and a two-vessel umbilical cord - are consistent with previously reported cases and support the vascular steal theory of pathogenesis.

Six findings identified in this case have not been previously identified in association with the sympus dipus variant in the reviewed literature, to our knowledge: lumbosacral myelomeningocele, Bochdalek diaphragmatic hernia, pyloric and duodenal atresia, rectal atresia, multiple subcapsular hepatic hematomas, and diminutive phallus. These associations suggest that the phenotypic spectrum of sympus dipus may be broader than previously recognized and that the underlying caudal developmental field defect may extend beyond the structures classically described.

Limitations of this report include the absence of maternal clinical history, lack of prenatal imaging data, unavailability of fetal organ histopathology, and inability to perform genetic evaluation. These factors should be systematically addressed in future autopsy protocols for sirenomelia cases.

This case contributes to the limited body of systematically documented sirenomelia autopsy cases from México and Latin America, and reinforces the critical role of complete fetal autopsy examination in characterizing the full extent of anomalies associated with this rare and lethal malformation. Future multicenter case series are needed to better define the phenotypic boundaries of each sirenomelia subtype.

## References

[1] Orioli IM, Amar E, Arteaga-Vazquez J (2011). Sirenomelia: an epidemiologic study in a large dataset from the International Clearinghouse of Birth Defects Surveillance and Research, and literature review. Am J Med Genet C Semin Med Genet.

[2] Stocker JT, Heifetz SA (1987). Sirenomelia. A morphological study of 33 cases and review of the literature. Perspect Pediatr Pathol.

[3] Valenzano M, Paoletti R, Rossi A, Farinini D, Garlaschi G, Fulcheri E (1999). Sirenomelia. Pathological features, antenatal ultrasonographic clues, and a review of current embryogenic theories. Hum Reprod Update.

[4] Escobar LF, Weaver DD, Bixler D, Hodes ME, Mitchell M (1987). Urorectal septum malformation sequence. Report of six cases and embryological analysis. Am J Dis Child.

[5] Duhamel B (1961). From the mermaid to anal imperforation: the syndrome of caudal regression. Arch Dis Child.

[6] Garrido-Allepuz C, Haro E, González-Lamuño D, Martínez-Frías ML, Bertocchini F, Ros MA (2011). A clinical and experimental overview of sirenomelia: insight into the mechanisms of congenital limb malformations. Dis Model Mech.

[7] Martínez-Frías ML, Bermejo E, Rodríguez-Pinilla E, Frías JL (2001). Exstrophy of the cloaca and exstrophy of the bladder: two different expressions of a primary developmental field defect. Am J Med Genet.

[8] Chaudhury N, Ghosh S, Saumondal B, Mandi S, Bose S (1984). Sirenomelia with multiple congenital anomalies. Indian Pediatr.

[9] Twickler D, Budorick N, Pretorius D, Grafe M, Currarino G (1993). Caudal regression versus sirenomelia: sonographic clues. J Ultrasound Med.

[10] Kurjak A, Kirkinen P, Latin V, Ivankovic D (1981). Ultrasonic assessment of fetal kidney function in normal and complicated pregnancies. Am J Obstet Gynecol.

[11] Castilla EE, Mastroiacovo P, López-Camelo JS, Saldarriaga W, Isaza C, Orioli IM (2008). Sirenomelia and cyclopia cluster in Cali, Colombia. Am J Med Genet A.

[12] Sosińska D, Gołębiewski A, Czauderna P (2024). Sirenomelia-challenges and treatment approach in a rare case. Birth Defects Res.

